# Detecting spatial-temporal cluster of hand foot and mouth disease in Beijing, China, 2009-2014

**DOI:** 10.1186/s12879-016-1547-6

**Published:** 2016-05-17

**Authors:** Haikun Qian, Da Huo, Xiaoli Wang, Lei Jia, Xitai Li, Jie Li, Zhiyong Gao, Baiwei Liu, Yi Tian, Xiaona Wu, Quanyi Wang

**Affiliations:** Beijing Center for Disease Prevention and Control, No.16 Hepingli Middle Street, Dongcheng District, Beijing, 100013 China

**Keywords:** Hand, foot, and mouth disease, Epidemiology, Space–time clustering, Spatial distribution, Spatiotemporal

## Abstract

**Background:**

The incidence of hand, foot, and mouth disease (HFMD) is extremely high, and has constituted a huge disease burden throughout Beijing in recent years. This study aimed to determine the spatiotemporal distribution and epidemic characteristics of HFMD.

**Methods:**

Descriptive statistics was used to analyze the data and estimate the epidemic peaks in 2009–2014. Space–time scanning detected spatiotemporal clusters and identified high-risk locations. Global and local Moran’s I statistics were used to measure the spatial autocorrelation. Geocoding was performed in ArcGIS, based on the present address codes of the patients and the centroids of the towns. Maps were created in ArcGIS to show the geographic spread of HFMD.

**Results:**

In total, 220,451probable cases of HFMD were reported in Beijing between January 2009 and December 2014: 12,749 (5.78 %) were laboratory confirmed, and 35 (0.02 %) were fatal. The median age of reported cases was 3.12 years (interquartile range 1.96–4.39). Coxsackievirus A16 (CV-A16), enterovirus 71 (EV-A71), and other enteroviruses accounted for 39.31, 35.36, and 25.33 % of the 12,749 confirmed cases, respectively. Many more severe cases were caused by EV-A71 (*χ*^2^ = 186.41, df = 1, *P* < 0.001) and other enteroviruses (*χ*^2^ = 156.44, df = 1, *P* < 0.001) than by CV-A16. A large single distinct peak occurred between May and July each year. Spatiotemporal clusters of HFMD were identified in Beijing during 2009–2014. The most likely clusters were detected and tended to move from the southwest (Fengtai and Daxing) southeastwards to Daxing and Tongzhou in 2009–2014. The incidence of HFMD was not randomly distributed, but showed global and local spatial autocorrelations.

**Conclusions:**

There were obvious spatiotemporal clusters of HFMD in Beijing in 2009–2014. High-incidence areas mainly occurred at the junctions of urban and rural zones. More attention should be paid to the epidemiological and spatiotemporal characteristics of HFMD to establish new strategies for its control. Health issues should be especially promoted in kindergartens and at urban–rural junctions.

## Background

Hand, foot, and mouth disease (HFMD) is an infectious disease caused by a number of enteroviruses, predominantly enterovirus A71 (EV-A71) and coxsackievirus A16 (CV-A16) [1]. As a self-limiting illness, typically including fever, skin eruptions on the hands and feet, and vesicles in the mouth, HFMD is common in children younger than 5 years, especially those aged 12–23 months [2]. However, HFMD is rare in adults and is typically mild when it occurs [3, 4]. The prevention and control of HFMD is very difficult, and several studies have found that the basic reproduction number of HFMD is larger than one corresponding to a rising incidence and is sensitive to asymptomatic infectious individuals and contaminated environments [5, 6]. Numerous large-scale outbreaks have been reported in Asia in the past two decades. In 1997, an outbreak of HFMD with 4253 cases and 41 deaths were reported in Malaysia [7]. In 1998, the largest EV-A71 epidemic reported 129,106 cases occurred and 78 died in Taiwan [8]. In March 2008, a large outbreak was reported in the city of Fuyang, Anhui Province in China, Of the 6049 cases reported, 3023 were hospitalized, 353 were severe, and 22 were fatal [9]. Around 490,000 infections and 126 deaths in children were reported in China in the same year [2]. Then the government listed HFMD as a notifiable Class C infectious disease in May 2008 and established a national enhanced surveillance system for HFMD, approximately 7.2 million probable cases were officially reported in 2008–2012 [10]. In Vietnam, more than 200,000 cases and 207 died were reported between 2011 and 2012 [11].

Many studies have explored the distribution of spatiotemporal clusters in the epidemiology of infectious diseases in recent years. Samphutthanon et al. used spatial-temporal analysis to explore the hotspots of HFMD in Northern Thailand [12]. Gui et al. investigated the epidemiological characteristics and high-incidence clusters of HFMD in Zhejiang, China, in 2008–2012 [13]. Wang et al. compared the outcomes of the local indicator of spatial association (LISA) and spatial filtering methods, and explored the spatial patterns of HFMD in Beijing in 2008–2012 [14]. However, HFMD was listed as a notifiable infectious disease in May, 2008, and the data from before this was installed may have been less reliable. The number of reported cases in 2008 was less than half the average number of the reported cases in the following 5 years. Moreover, when the HFMD data for an additional 2 years (2013 and 2014) were added to this study, more clusters were distributed in Beijing.

Based on the surveillance data for Beijing in 2009 to 2014, epidemiological and space–time scan statistics on different spatial scales and a spatial autocorrelation analysis were conducted in this study to detect the spatiotemporal clusters and characterize the epidemiological features of HFMD. Based on the results, appropriate regional public-health intervention strategies can be formulated to prevent and control HFMD outbreaks.

## Methods

### Case definitions

A “probable case” of HFMD was defined as a patient with vesicular rash on the hands, mouth, feet, or buttocks, with or without fever. A “laboratory-confirmed case” was defined as a probable case with laboratory evidence of HFMD based on nucleic acid amplification, virus isolation, or the detection of neutralizing antibodies (against CV-A16, EV-A71, or other enteroviruses that cause HFMD). The probable and confirmed cases were classified as severe if the patient had any neurological complications or cardiopulmonary complications; otherwise, the patient was classified as a mild case. These are the same definitions as used by the national surveillance system in China. Children aged <3, 3–6, and 6–14 years were defined as scattered children, kindergarten children, and students, respectively.

### Data collection

Probable and confirmed cases were reported by physician online, using a standardized form that included the basic demographic information, case classification, severity, date of symptom onset, date of diagnosis, date of death, virus serotype, and code of district. In this study, research data were collected between 2009 and 2014 with the National Infectious Disease Information Management System (China).

Beijing, the capital of China, has a population exceeding 20 million residents, and consists of 16 districts divided into 309 towns. The demographic data for each town were based on the 2010 census data published in the Beijing Statistical Yearbook (http://www.bjstats.gov.cn/). The Beijing map is purchased from the Beijing Institute of Surveying and Mapping and used with permission. The home addresses of the HFMD cases were matched to the geographic coordinates of the towns.

### Statistical analysis and visualization

Descriptive statistics were used to describe the epidemiological features of HFMD, and were prepared and analyzed with the software R 3.2.3 (https://www.r-project.org/). Mapping was performed in ArcGIS 10.1 (https://www.arcgis.com/), based on the present address code of the case and the centroid of the town of residence. The incidence, based on the whole population, and the geographic distribution are presented according to town.

### Space–time scan statistic

The space–time scan statistic is defined by a cylindrical window with a circular geographic base and a height corresponding to time. As the window moves in space and time, an infinite number of overlapping cylinders of different size and shapes appear, jointly covering the entire study region, where each cylinder reflects a potential cluster. For each location and size of the window, the alternative hypothesis is that there is an elevated risk within the window compared with the risk outside the window. The relative risk can be calculated with the ratios of the observed number of cases to the expected number of cases inside and outside the window. The log likelihood ratio (LLR) is calculated with a likelihood function using the formula given in the next paragraph. The most likely cluster is the one with the maximum LLR. The *P* value is obtained with Monte Carlo hypothesis testing, with 999 simulations [15].

Under the Poisson assumption, the likelihood function for a specific window is proportional to:

$$ {\left(\frac{c}{E\left[c\right]}\right)}^c{\left(\frac{C-c}{C-E\left[c\right]}\right)}^{C-c}I\left(\right) $$

where *C* is the total number of cases, *c* and *E*[*c*] are the observed and expected number of cases, respectively, within the window under the null hypothesis, and *I* () is equal to 1, as an indicator function for clusters with high rates.

In this study, the retrospective space–time statistic was calculated to detect the spatiotemporal clusters with SaTScan 9.1.1 (http://www.satscan.org/). A discrete Poisson model was used with the latitude/longitude coordinates. The maximum spatial cluster size on the spatial windows tab was specified in kilometers. The maximum temporal cluster size is specified in terms of days. Based on the HFMD incubation period of 2–10 days, with an average of 3–5 days [16–19], the maximum temporal cluster size was set to 7 days. Maximum spatial cluster sizes of 5–10 km and 15–20 km were specified to identify the shifts of the clusters each year.

### Spatial autocorrelation analysis

Spatial autocorrelation statistics are a spatial method used to measure and analyze the degree of dependence among observations in a geographic space. Global and local Moran’s *I* statistics are used to measure spatial autocorrelation. Global Moran’s *I* is used to test for clustering and to visualize the data with a Moran scatter plot, in which the slope of the regression line corresponds to Moran’s *I* [20]. Local Moran’s *I* statistic calculated with LISA was used to test for clusters in the significant spatial autocorrelation regions and to visualize them in the form of significance and cluster maps. Four patterns of spatial correlation are identified by LISA, high–high, low–low, high–low, and low–high clusters. For instance, in the “high–high” clusters, areas with high values of a variable are surrounded by neighboring areas with high values, and so forth. In fact, the high–high pattern indicates a hot spot, and is most meaningful pattern for the purposes of disease control and prevention. Spatial autocorrelation statistics require the measurement of a spatial weights matrix, which reflects the intensity of the geographic relationship between the observations in a neighborhood. In this study, the spatial weights were used to describe the spatial relationships among counties in Beijing and were created with the queen contiguity rule. The significance of Moran’s *I* was validated by Monte Carlo tests with Z statistics, and the *P* values were based on a permutation test. Spatial autocorrelation with high–high is present if Moran’s *I* is larger than zero, with statistical significance. For the sensitivity analysis, the number of permutations can be changed from 99 to 9999, and then the significance cutoff value changes. The formula for global Moran’s *I* is:

$$ \begin{array}{c}\hfill I=\frac{n{\displaystyle \sum_{i=1}^n{\displaystyle \sum_{j=1}^n{w}_{ij}\left({x}_i-\overline{x}\right)\left({x}_j-\overline{x}\right)}}}{{\displaystyle \sum_{i=1}^n{\displaystyle \sum_{j=1}^n{w}_{ij}}}{\displaystyle \sum_{i=1}^n{\left({x}_i-\overline{x}\right)}^2}}=\frac{{\displaystyle \sum_{i=1}^n{\displaystyle \sum_{j\ne 1}^n{w}_{ij}\left({x}_i-\overline{x}\right)\left({x}_j-\overline{x}\right)}}}{S^2{\displaystyle \sum_{i=1}^n{\displaystyle \sum_{j=1}^n{w}_{ij}}}}\hfill \\ {}\hfill \begin{array}{cc}\hfill Z=\frac{Moran's\kern0.5em I-E(I)}{\sqrt{VAR(I)}}\hfill & \hfill E(I)=-\frac{1}{n-1}\hfill \end{array}\hfill \end{array} $$

where *n* is the number of districts, *i* and *j* are two different districts, *x*_*i*_ and *x*_*j*_ are the values of the observed indicators (incidence) for districts *i* and *j*, $\overline{x}$ is the average of the indicators of all districts, and *w*_*ij*_ is the spatial weight between *i* and *j*. The formula is the same for local Moran’s *I*, except that *i* and *j* refer to local counties [21]. GeoDa1.6.7 (https://geodacenter.asu.edu/software/downloads) was used to conduct the spatial autocorrelation analyses.

### Ethics statement

This retrospective study was approved by the Ethics Committee of the Beijing Center for Disease Prevention and Control. All data (including name, identity information, address, telephone number, etc.) were anonymized, and no individual information can be identified.

## Results

### Epidemiological features

In total, 220,451 probable cases of HFMD were reported in Beijing from January 2009 to December 2014, of which 12,749 (5.78 %) were laboratory confirmed and 35 (0.02 %) were fatal. The age-specific (<5 years) incidence rate of HFMD markedly increased each year, with 261.68 per 10,000, 472.37 per 10,000, and 538.65 per 10,000 in 2009, 2010, and 2011, respectively. The incidence then decreased (442.93 per 10,000 in 2012, 375.10 per 10,000 in 2013), but peaked at 494.26 per 10,000 in 2014 (Table 1).

**Table 1:** Epidemiological features of HFMD in Beijing, China, 2009–2014

	2009 (*n* = 24,483)		2010 (*n* = 45,409)		2011 (*n* = 30,843)		2012 (*n* = 38,528)		2013 (*n* = 33,763)		2014 (*n* = 47,425)	
	Cases (%)	Incidence (1/10,000)	Cases (%)	Incidence (1/10,000)	Cases (%)	Incidence (1/10,000)	Cases (%)	Incidence (1/10,000)	Cases (%)	Incidence (1/10,000)	Cases (%)	Incidence (1/10,000)
Age group												
0-	1530 (6)	94.52	2626 (6)	157.09	1593 (5)	158.56	2055 (5)	172.59	2735 (8)	225.05	1759 (4)	107.50
1-	2801 (12)	170.60	9661 (21)	566.58	6203 (20)	602.70	7466 (20)	483.01	8525 (25)	536.01	9725 (21)	777.83
2-	6390 (26)	397.23	10,216 (23)	606.90	6573 (21)	652.70	7437 (19)	484.64	5839 (17)	366.65	9052 (19)	547.16
3-	5883 (24)	387.97	10,209 (23)	637.89	7435 (24)	776.58	8610 (22)	586.46	6285 (19)	409.21	9954 (21)	634.32
4-	3906 (16)	268.95	6033 (13)	391.30	4580 (15)	509.00	6147 (16)	432.54	4473 (13)	299.71	7248 (15)	475.25
5-	2031 (8)	144.32	3192 (7)	213.44	2068 (7)	237.79	3163 (8)	339.64	2466 (7)	253.45	4214 (9)	425.28
6-	1304 (5)	20.44	2294 (5)	32.45	1572 (5)	39.86	2648 (7)	57.42	2052 (6)	39.78	4004 (8)	75.71
10-	369 (2)	3.05	614 (1)	5.02	449 (2)	6.88	520 (2)	10.36	573 (2)	11.47	687 (1)	13.52
15-	269 (1)	0.19	564 (1)	0.38	370 (1)	0.21	482 (1)	0.26	815 (3)	0.43	782 (2)	0.41
Gender												
Male	14,826 (61)	17.37	27,373 (60)	30.98	18,474 (60)	18.27	23,079 (60)	22.18	20,392 (60)	19.09	27,985 (59)	25.66
Female	9657 (39)	11.48	18,036 (40)	20.70	12,369 (40)	13.02	15,449 (40)	15.80	13,371 (40)	13.35	19,440 (41)	18.98
Gender ratio	1.54:1	–	1.52:1	–	1.49:1	–	1.49:1	–	1.53:1	–	1.44:1	–
Occupation												
Scattered children^a^	13,552 (55)	278.32	23,474 (52)	463.87	15,563 (50)	511.75	19,840 (51)	464.53	19,198 (57)	436.49	23,694 (50)	521.79
Kindergarten children^a^	9597 (39)	219.31	19,330 (42)	416.80	13,511 (44)	495.47	16,090 (42)	421.14	11,967 (35)	299.08	19,971 (42)	488.86
Students^a^	1133 (5)	6.14	2184 (5)	11.32	1486 (5)	14.19	2220 (6)	23.05	1934 (6)	19.04	3107 (7)	29.96
Other	201 (1)	0.14	421 (1)	0.29	283 (1)	0.16	378 (1)	0.21	664 (2)	0.35	653 (1)	0.34

In occupation, ^a^, Children under 3, 3–6, and 6–14 years old are defined as scattered children, kindergarten children and students respectively. Therefore, the incidence rates of those children are calculated as the number of cases divided by the population of corresponding age group

Most patients were children younger than 5 years, accounting for the largest proportion of all reported cases (ranging from 88.47 to 93.55 %). The median age of reported cases was 3.12 years (interquartile range [IQR] 1.96–4.39). The incidences of HFMD in the 1-, 2-, and 3-year age groups were higher than in the other year groups. The incidence of HFMD in children over 6 years old decreased with increasing age. The gender-specific (<5 years) incidence rate was far higher in male (489.38 per 10,000) than in female (352.47 per 10,000), and the ratio of male to female patients was about 1.50:1. The median age of the male patients was 3.14 years (IQR 1.97–4.41), and that of the female patients was 3.08 years (IQR 1.95–4.34). Scattered children accounted for the largest of proportion of cases based on occupation, followed closely by kindergarten children (Table 1).

**Fig. 1:**
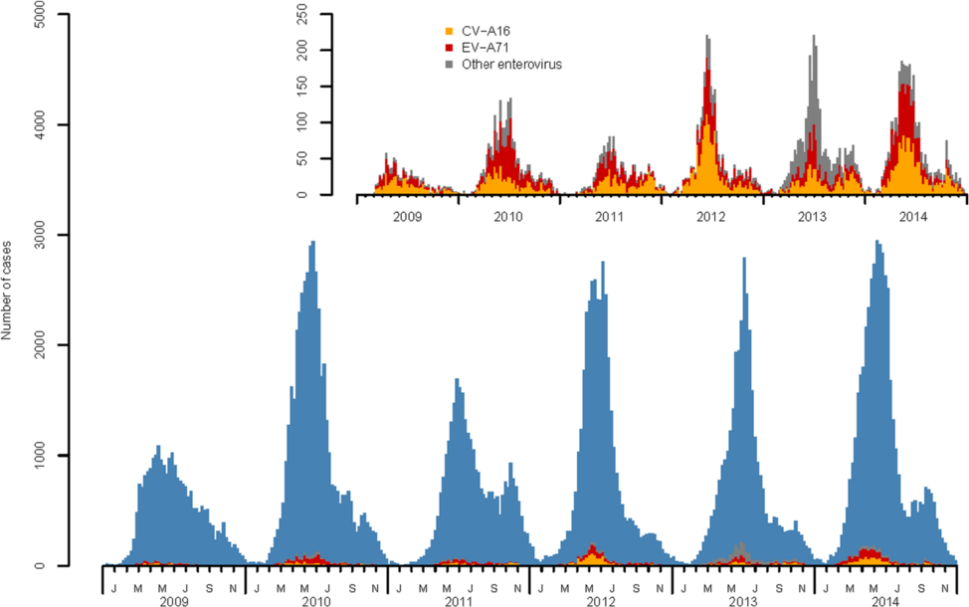
Weekly reported cases of HFMD in Beijing, China, 2009–2014^*^. ^*^ The insert is distributions of EV-A71, CV-A16 and other enteroviruses of HFMD from 2009 to 2014

CV-A16, EV-A71, and other enteroviruses accounted for 39.31, 35.36, and 25.33 % of the 12,749 laboratory-confirmed cases, respectively. The predominant serotypes were CV-A16 (2009, 2011–2012, 2014) and EV-A71 (2010 and 2011). Other enteroviruses were the predominant etiological agents in 2013 (Table 2 and Fig. 1). The pathogens differed between the mild and severe cases. Of the 573 confirmed severe cases, EV-A71 accounted for 52.88 % (303 cases), CV-A16 for 11.34 % (65 cases), and other enteroviruses for 35.78 % (205 cases). Many more severe cases were caused by EV-A71 (*χ*^2^ = 186.41, df = 1, *P* < 0.001) and other enteroviruses (*χ*^2^ = 156.44, df = 1, *P* < 0.001) than by CV-A16.

**Table 2:** Pathogen of HFMD in Beijing, China, 2009–2014

Pathogen	2009	2010	2011	2012	2013	2014
CV-A16	459 (52)	626 (30)	607 (43)	1283 (53)	731 (25)	1305 (42)
EV-A71	316 (36)	988 (48)	594 (42)	824 (34)	628 (22)	1158 (38)
Other EV	107 (12)	447 (22)	203 (15)	319 (13)	1546 (53)	608 (20)
Total	882 (100)	2061 (100)	1404 (100)	2426 (100)	2905 (100)	3071 (100)

**Fig. 2:**
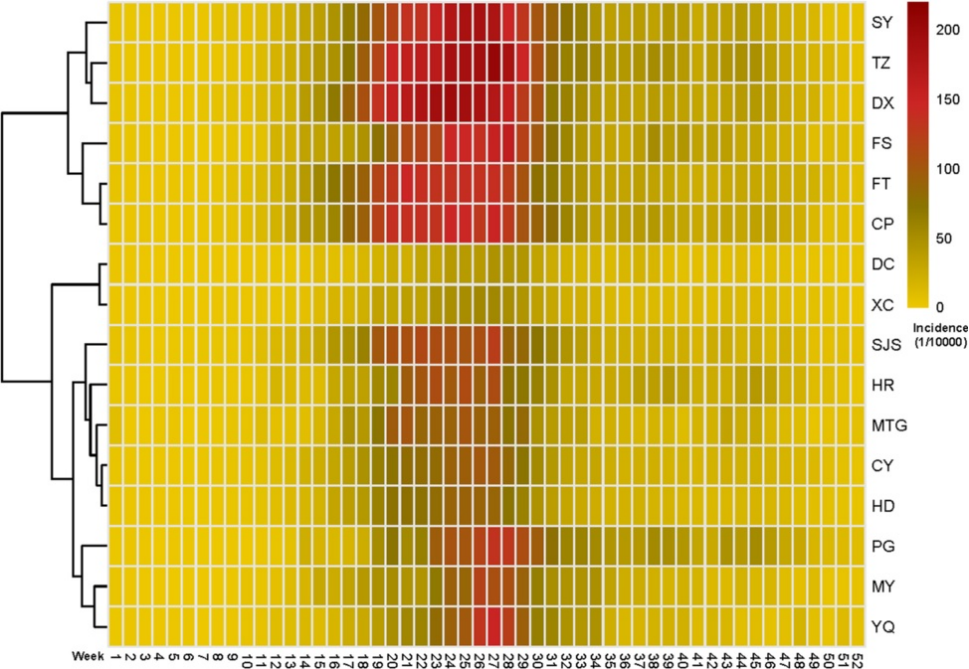
Heat map of surveillance data for HFMD by county of Beijing, 2009–2014

**Fig. 3:**
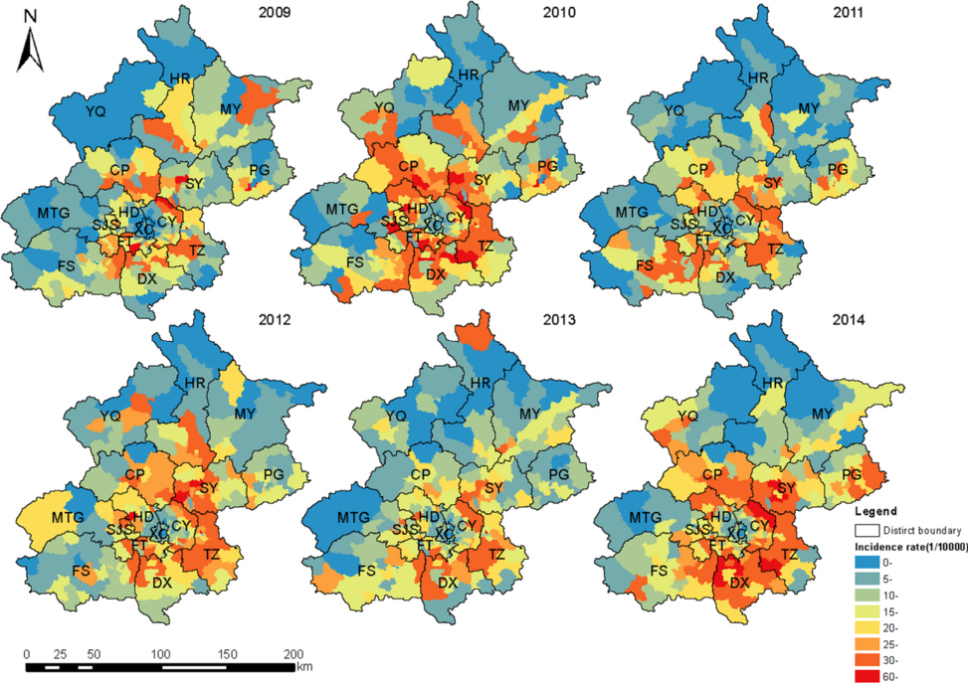
Distribution of incidence for HFMD at township level in Beijing, China, 2009–2014. The Beijing map was purchased from the Beijing Institute of Surveying and Mapping and used with permission

A major single peak in HFMD occurred between May and July each year, followed by a minor peak observed between October and November (Fig. 1). A heat map based on the weekly surveillance data for HFMD showed that the annual peak was observed from week 18 (From 29th April to 2nd May) to the end of week 30 (From 22nd to 28th July), lasting about 13 weeks (about 3 months). Two main geographic regions with similar incidences were identified (Fig. 2). The distribution of incidence was also mapped at the town level. The incidence densities of HFMD were much higher in 2010, 2012, and 2014 than in the other years. The incidence was higher in some areas of the suburbs. In particular, the incidence of HFMD formed a ring-like distribution between urban and rural areas (Fig. 3).

### Spatiotemporal distribution analysis

Statistically significant spatiotemporal clusters were detected in 2009–2014. With a maximum scanning radius of 5 km, the most likely clusters were located in the FT district annually from 2009 to 2011. The cluster centers were at 39.85N, 116.28E, and the cluster radii were around 4.70–4.92 km. However, in 2012–2014, the most likely clusters located in DX shifted from north to south, with cluster centers at 39.76N, 116.33E and cluster radii of around 3.76 km.

To detect more clusters, the scanning radius was gradually increased. The same analysis was performed with a maximum spatial cluster scanning radius of 10 km. The most likely clusters were mainly located in FT and a few towns in SJS in 2009 and 2011. The cluster centers were at 39.83N, 116.23E, and the cluster radii were 7.22 km in 2009 and 9.73 km in 2011. The most likely clusters were located in FT and DX in 2010, 2012–13, and 2014. The cluster centers were in the border region between FT and DX, and the cluster radius was almost 10 km each year. Similarly, with a maximum spatial cluster scanning radius of 15 km, the most likely clusters were detected predominantly in FT (2009, 2011, and 2013) and DX (2010, 2012, and 2014). These results indicate that the incidence of HFMD in the clusters was distributed abnormally. When a maximum cluster radius of 20 km was used, the most likely clusters were detected in 2009 and 2011. The areas mainly included some town streets at the junctions of three districts FT, DX, and FS. The most likely cluster in 2011 was larger than that in 2009, but in 2010, 2012–2013, and 2014, the most likely clusters changed and were mainly distributed at the junctions of FT, TZ, CY, and DX.

**Fig. 4:**
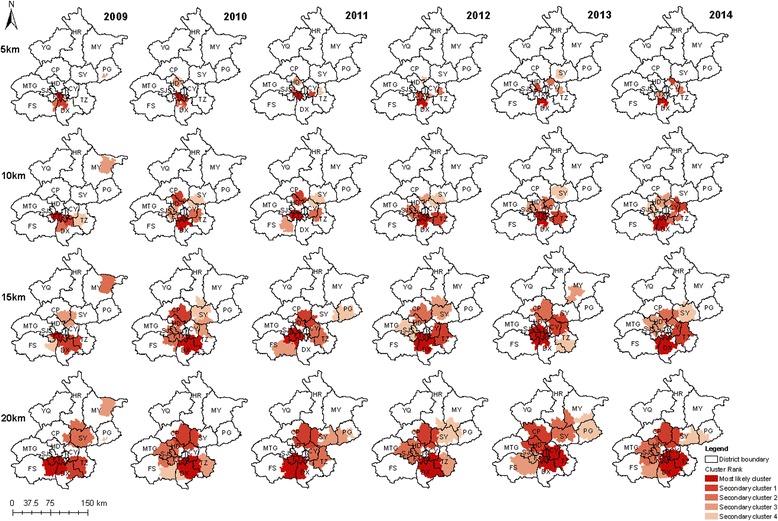
Spatial-temporal clusters of HFMD in Beijing, China, 2009–2014. Spatial cluster radius was from 5 to 20 km, temporal cluster size was set to 7 days. The Beijing map was purchased from the Beijing Institute of Surveying and Mapping and used with permission

With different spatial radii, secondary clusters were detected that were less likely than the most likely clusters. These clusters were mainly distributed at the junctions of urban and rural districts (Table 3 and Fig. 4).

**Table 3:** The clusters of HFMD in Beijing, China, 2009–2014 by Spatial-temporal Scan ^a^

Year	Spatial radius (5–20 km)	Date of cluster	Hotspot districts	Number of cases	Expected cases	Relative risk	Log likelihood ratio	*P*-value
2009	5 km	17–23 May	FT	179	20	9.02	233.96	<0.001
	10 km	17–23 May	FT	229	26	8.78	293.75	<0.001
	15 km	17–23 May	FT	229	26	8.78	293.75	<0.001
	20 km	18–24 May	FT, FS, DX	296	47	6.31	294.81	<0.001
2010	5 km	24–30 Apr	FT	241	37	6.52	247.3	<0.001
	10 km	12–18 June	FT, DX	478	74	6.48	487.07	<0.001
	15 km	21–27 June	FT, DX, TZ, CY	578	93	6.30	574.67	<0.001
	20 km	12–18 June	FT, DX, TZ, CY	808	157	5.21	676.09	<0.001
2011	5 km	21–27 June	FT	212	27	7.99	254.53	<0.001
	10 km	19–25 June	FT, SJS	315	50	6.34	315.12	<0.001
	15 km	18–24 June	FT, SJS, FS	382	68	5.68	346.92	<0.001
	20 km	18–24 June	FT, SJS, FS, DX	520	94	5.62	467.43	<0.001
2012	5 km	1–7 June	DX	168	18	9.50	227.58	<0.001
	10 km	23–29 May	DX, FT, CY	510	90	5.74	467.79	<0.001
	15 km	23–29 May	DX, FT, FS, CY	695	131	5.38	599.01	<0.001
	20 km	23–29 May	DX, FT, CY, TZ	851	173	5.00	681.99	<0.001
2013	5 km	1–7 July	DX,	139	16	8.87	179.86	<0.001
	10 km	1–7 July	DX, FT	441	89	4.99	354.06	<0.001
	15 km	1–7 July	DX, FT, SJS, HD, FS	811	172	4.79	623.15	<0.001
	20 km	1–7 July	DX, FT, DC, XC, CY, TZ	1240	319	4.00	774.89	<0.001
2014	5 km	10–16 June	DX	256	22	11.46	390.01	<0.001
	10 km	10–16 June	DX, FT, FS	368	38	9.63	502.59	<0.001
	15 km	24–30 May	DX, FT, FS	480	59	8.15	584.04	<0.001
	20 km	21–27 June	DX, FT, DC, XC, CY, TZ	1159	320	3.68	659.01	<0.001

^a^The maximum temporal cluster size was set to 7 days

**Fig. 5:**
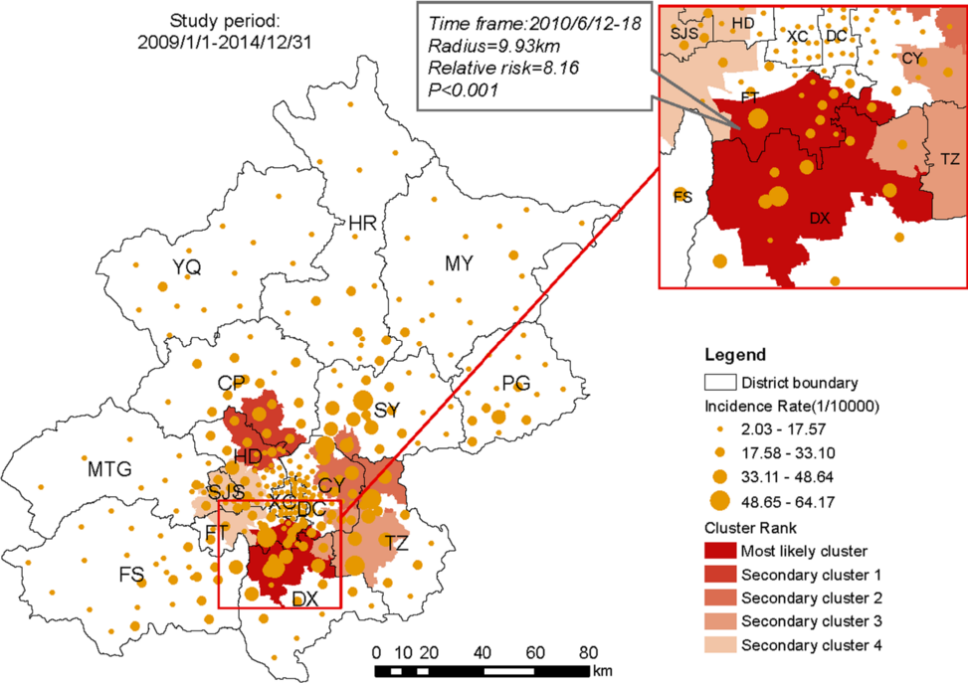
Spatial-temporal clusters of HFMD in Beijing, China, 2009–2014. Spatial cluster size was a circle with a 10 km radius, temporal cluster size was set to 7 days. The Beijing map was purchased from the Beijing Institute of Surveying and Mapping and used with permission

A space–time scan was also conducted with a 10 km radius for the whole study period. The most likely cluster and 38 secondary clusters were identified with statistical significance in 2009–2014, with a relative risk of 8.16, and a *P* value < 0.001 (Fig. 5). The most likely cluster included 16 towns mostly located in the southern part of FT and the northern part of DX. The cluster center was at 39.77N, 116.37E and the cluster radius was 9.93 km.

### Hotspot detection and analysis

**Fig. 6:**
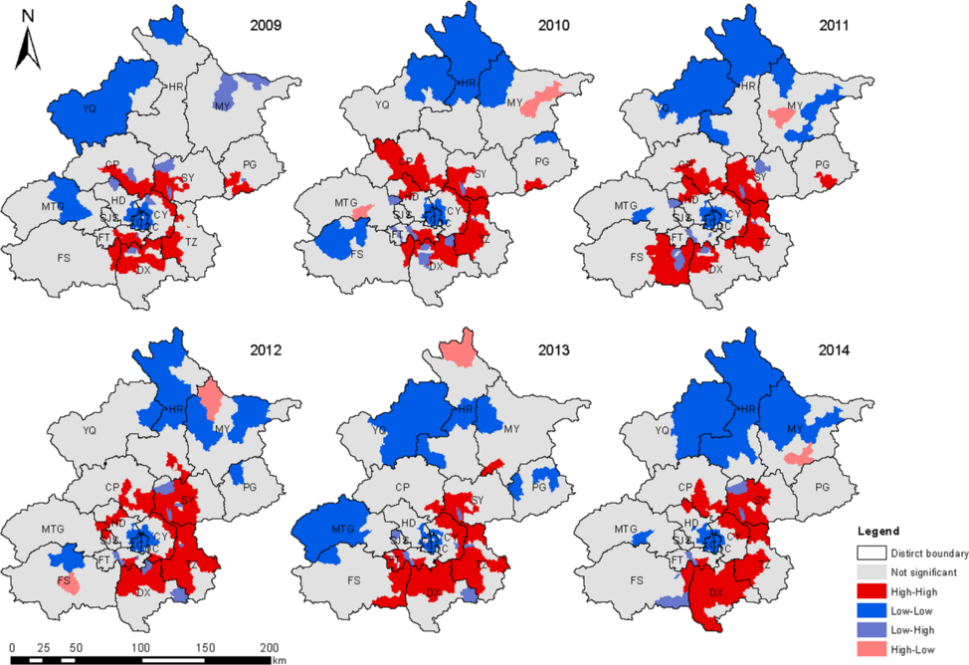
The LISA cluster map of HFMD in Beijing, China, 2009–2014. The Beijing map was purchased from the Beijing Institute of Surveying and Mapping and used with permission

The global and local spatial autocorrelations of the incidence of HFMD in Beijing in 2009–2014 were analyzed with a spatial autocorrelation analysis. The incidence of HFMD was not randomly distributed. In the global spatial autocorrelation analysis, Moran’s *I* was statistically significant and ranged from 0.32 to 0.51 (Table 4). A LISA map of HFMD clusters was used to illustrate the local spatial autocorrelation at the town level (Fig. 6). The high–high clusters, or hotspots (red color), were predominantly in southern districts, eastern districts, and northern districts, including DX, TZ, SY, and CP, and occurred in an inverted letter “C” around central Beijing. Visual inspection clearly showed that the incidence of HFMD was high at the junctions of urban and rural areas and in neighboring areas, whereas in the outer suburbs (YQ, HR, and MY) and old cities (DC and XC), the incidences of HFMD was low, with low–low clusters (blue color). The other districts had high–low or low–high clusters or nonsignificant (no clusters) areas.

**Table 4:** The Moran’s *I* of global spatial autocorrelation analysis for HFMD during 2009–2014

Year	Moran’s *I*	E[*I*]	Mean	Standard Deviation	*Z* score	*P*-value
2009	0.32	−0.0032	−0.0027	0.0336	9.52	<0.001
2010	0.37	−0.0032	−0.0035	0.0350	10.69	<0.001
2011	0.43	−0.0032	−0.0025	0.0353	12.35	<0.001
2012	0.45	−0.0032	−0.0040	0.0347	13.21	<0.001
2013	0.41	−0.0032	−0.0036	0.0338	12.35	<0.001
2014	0.51	−0.0032	−0.0021	0.0346	14.70	<0.001

## Discussion

In this study, most cases of HFMD were in children younger than 5 years. It is noteworthy that the median age of the reported cases was 3.12 years. These findings are consistent with those of other studies [10, 22, 23]. Close contact with HFMD patients and poor personal hygiene are also risk factors for HFMD [4]. Most children (between 3 and 6 years old) receive preschool education in kindergartens in Beijing and are therefore in close contact with each other, increasing their chance of infection above that of younger children.

This study reconfirmed that CV-A16 and EV-A71 were the most common etiological pathogens of HFMD in Beijing, but that EV-A71 and other enteroviruses were closely associated with severe cases. Infections with other enterovirus were also particularly predominant in 2013 and accounted for a large proportion of severe cases in Beijing from 2009 to 2014. These findings indicate that other enteroviruses are also potential threats and must be monitored, so the other enteroviruses causing HFMD in Beijing must be classified [24]. Outbreaks of HFMD associated with other enteroviruses, such as CV-A6 and CV-A10, have been described in France [25], Finland [26], and most recently in Japan [27]. CV-A6 was predominant in outbreaks reported in China in 2013 [28–30], and is associated with a more severe and profound course of the disease, affecting both children and adults. The involvement of a more virulent strain of enterovirus should be suspected if an adult patient presents with a clinical diagnosis of HFMD [31].

As in other studies, a significant increase in the incidence of HFMD was observed as a large distinct single peak between May and July in Beijing every year (Figs. 1 and 2). The annual peak in HFMD occurs in June in north China [10], but was more prevalent between May and July in Beijing [32]. This discrepancy may be related to meteorological factors (high average temperature and high relative humidity) [18, 33]. The incidence of HFMD increases with increasing temperatures, with the greatest association at 25.0–27.5 °C [32]. In this study, spatiotemporal scans were performed with different radii in the same years. The most likely cluster and secondary clusters were identified, and became larger as the radii increased. However, the radii only weakly influenced the center of the cluster and the date of the cluster. In other words, the centers and time frames of the clusters showed little or no change with different radii. However, a shift was detected in which the most likely cluster of HFMD tended to move from FT and DX southeastwards to DX and TZ from year to year. During the whole study period, the most likely cluster was basically detected with a 10 km radius in FT and DX, together with 38 secondary clusters mainly located in the zones between urban and rural areas. The global spatial autocorrelation showed that the incidence of HFMD in Beijing in 2009–2014 was not randomly distributed. The hot spots, or high-incidence areas, were most often found at the junctions of urban and rural areas, whereas the outer suburbs and old cities had low incidences on the LISA map. Both the spatiotemporal scan and spatial autocorrelation analyses showed that the clusters with high HFMD incidence occurred at the junctions of urban and rural areas, and that the incidence was low in the outer suburbs and old cities. The distribution of HFMD presented as a ring-like or inverted “C” zone around old central Beijing. These hot-spots are built-up areas with poor living conditions and poor sanitation, ever-growing floating high-density populations, and poor quality of kindergartens [34, 35]. All these risk factors maybe lead to a higher incidence of HFMD [36]. Two main geographic regions with high or low incidences clustered together and were identified on a heat map. The first high-incidence region included new developing urban areas (DX, TZ, SY, and CP) and the second low-incidence included SJS, HR, MTG, CY, HD, PG, MY, and YQ. The heat map also showed that the annual peak extended from week 18 to the end of week 30, lasting for about 13 weeks (about 3 months), consistent with the findings of the present study.

The present study had several limitations. First, it may have underestimated the number of HFMD cases. As a self-limiting illness, patients with HFMD may not attend hospital, which makes them untraceable by the surveillance system. Second, younger children are susceptible to HFMD, but in this study, both the spatiotemporal scan and the spatial autocorrelation analysis were based on the incidence in the whole population, as in other studies [14, 37, 38], rather than for specific age groups. This could have biased the clusters. Finally, model uncertainty means that many findings can often be overturned by small changes in the model specifications [39]. The scan statistic determined with SaTScan is the most commonly used cluster-detection method and has been applied broadly [40]. The results of spatiotemporal scans are sensitive to various parameters, so the selection of parameters is very important. It is noteworthy that in SaTScan, the maximum circle size parameter can be either the percentage of the total population at risk or the geographic size of the circle. The default maximum spatial cluster size value in the SaTScan user guide [15] is 50 % of the population at risk, and with this setting, the primary clusters often cover a large proportion of the study area and seldom produce informative results. The results can also vary significantly, depending upon the levels of the spatial scales [41, 42].

## Conclusions

In summary, the occurrence of HFMD is widespread around Beijing, especially among children under 5 years old, and the median age of reported cases was 3.12 years. A major single peak occurred between May and July annually, followed by a minor peak between October and November. CV-A16 and EV-A71 were the most common etiological pathogens of HFMD in Beijing, although other enteroviruses, such as CV-A6 and CV-A10, must be afforded more attention in future studies. This study reports the spatiotemporal distribution of HFMD clusters in Beijing. The most likely cluster was detected in the southwest (FT and DX) and tended to move southeastwards (towards DX and TZ) in Beijing in 2009–2014. The incidence of HFMD was not randomly distributed, with global and local spatial autocorrelations. Hot spots, or high-incidence areas, mainly occurred at the junctions of urban and rural zones. New strategies and methods must be developed and more attention paid to the epidemiological and spatiotemporal characteristics of HFMD so that control measures can be improved. For instance, health promotion campaigns should focus on kindergartens and the junctions between urban and rural zones.

### Ethics approval and consent to participate

This retrospective study was approved by the Ethics Committee of the Beijing Center for Disease Prevention and Control. All data (including name, identity information, address, telephone number, etc.) were anonymized, and no individual information can be identified.

### Availability of data and materials

Data is available to all researchers upon request.

## Abbreviations

CV-A10: Coxsackievirus A10
CV-A16: Coxsackievirus A16
CV-A6: Coxsackievirus A6
CP: Changping
CY: Chaoyang
DC: Doncheng
df: degree of freedom
DX: Daxing
EV-A71: Enterovirus A71
FS: Fangshan
FT: Fentai
HD: Haidian
HFMD: Hand, foot and mouth disease
HR: Huairou
IQR: Inter-quartile range
MTG: Mentougou
MY: Miyun
Other EV: other enteroviruses
PG: Pinggu
SJS: Shijingshan
SY: Shunyi
TZ: Tongzhou
XC: Xicheng
YQ: Yanqing
